# Cryptococcosis in Pediatric Renal Transplant Recipients: Comparative Insights from Adult Cases

**DOI:** 10.3390/medicina61061108

**Published:** 2025-06-18

**Authors:** Guido Gembillo, Chiara Terzo, Salvatore Silipigni, Luca Soraci, Emmanuele Venanzi Rullo, Ylenia Russotto, Chiara Casuscelli, Maria Elsa Gambuzza, Maria Princiotto, Lorenzo Lo Cicero, Luigi Peritore, Concetto Sessa, Domenico Santoro

**Affiliations:** 1 Unit of Nephrology and Dialysis, Department of Clinical and Experimental Medicine, University of Messina, 98125 Messina, Italy; chiaraterzo96@gmail.com (C.T.); chiara.casuscelli88@gmail.com (C.C.); lorenzolocicero95@gmail.com (L.L.C.); luigiperitore1994@gmail.com (L.P.); dsantoro@unime.it (D.S.); 2 Interventional Radiology Unit, Department of Biomedical and Dental Sciences and Morphological and Functional Imaging, University of Messina, A.O.U. Policlinico ‘G. Martino’, 98125 Messina, Italy; salvo.sili@gmail.com; 3 Unit of Geriatric Medicine, Italian National Research Center on Aging (IRCCS INRCA), 87100 Cosenza, Italy; l.soraci@inrca.it; 4 Unit of Infectious Disease, Department of Clinical and Experimental Medicine, University of Messina, 98100 Messina, Italy; evenanzirullo@unime.it (E.V.R.); ylenia.russ@gmail.com (Y.R.); 5 Territorial Office of Messina, Ministry of Health, 98100 Messina, Italy; me.gambuzza@sanita.it; 6 Laboratory of Pharmacoepidemiology and Biostatistics, Italian National Research Center on Aging (IRCCS INRCA), 87100 Cosenza, Italy; mariaprinciotto98@gmail.com; 7 Nephrology and Dialysis Department, Maggiore “Nino Baglieri” Hospital, 97015 Modica, Italy; concettosessa@gmail.com

**Keywords:** *Cryptococcus*, chronic kidney disease, infectious disease, pediatry, imaging, transplantation, liver, lungs, pulmonary, cutaneous localization, central nervous system, neurological involvement, neurology

## Abstract

Cryptococcosis, an opportunistic fungal infection predominantly caused by *Cryptococcus neoformans*, is the third most common invasive fungal disease in solid organ transplant recipients. While well-characterized in adult kidney transplant (KT) patients, pediatric data remain sparse. This article compares clinical presentation, immune response, renal involvement, and management strategies of cryptococcosis between adult and pediatric KT recipients. In adults, the disease typically presents as cryptococcal meningitis or pulmonary infection, often complicated by delayed diagnosis and high mortality. In contrast, children frequently exhibit non-specific respiratory symptoms or disseminated disease, reflecting immune immaturity and increased susceptibility to hematogenous spread. Key immunopathological differences include impaired Th1 type responses, macrophage dysfunction, and variable complement activity across age groups. Management involves similar antifungal regimens such as liposomal amphotericin B, flucytosine, and fluconazole, but requires weight-based dosing and careful toxicity monitoring in pediatric patients. Early diagnosis through serum cryptococcal antigen screening, appropriate adjustment of immunosuppressive therapy, and coordinated multidisciplinary care are essential. The findings underscore the need for pediatric specific research and clinical vigilance, emphasizing tailored antifungal dosing and individualized immune management to improve outcomes in this vulnerable population.

## 1. Introduction

Fungal infections occur in 15–42% of organ transplant recipients [1] and cryptococcosis remains a significant threat to solid organ transplant (SOT) recipients, ranking as the third most common invasive fungal disease in this population [2]. Despite being frequently reported in transplant recipients, cryptococcosis is not the most common invasive fungal infection (IFI), as reported by the TRANSNET results who investigated IFIs in transplant recipients from 2001 to 2006 and in which cryptococcosis accounted for 8% of IFIs, while invasive candidiasis represented the most common with a percentage of 53% [3].

In kidney transplant (KT) recipients, the infection is often caused by *Cryptococcus neoformans* and, less frequently, by *Cryptococcus gattii*, which primarily affects the respiratory system [4]. Immunosuppression plays a central role in disease development, as both pediatric and adult KT recipients are at risk due to T cell depletion therapies and prolonged use of immunosuppressants [5].

Calcineurin inhibitors have in vitro antifungal effects, reducing the likelihood of disseminated fungal infections in transplant recipients [6].

Although cryptococcosis has been extensively described in adult transplant patients, pediatric data remain scarce, necessitating further research into pediatric-specific risk factors and disease progression. Pediatric patients often exhibit different clinical presentations, and their response to infection may be influenced by age-related immune system differences. The latest European Confederation of Medical Mycology (ECMM) and International Society for Human and Animal Mycology (ISHAM) guidelines stress the importance of early detection using serum cryptococcal antigen (CrAg) testing in transplant recipients at risk of cryptococcal disease [7]. This narrative review aims to highlight key similarities and differences between adult and pediatric KT patients with cryptococcosis, providing an integrated approach to diagnosis and treatment.

## 2. Clinical Manifestations: Adult vs. Pediatric Patients

Cryptococcus infection can range from asymptomatic colonization to disseminated disease, depending on the immunological condition of the host. The central nervous system (CNS) is the main target of Cryptococcus, but when acquired it can spread to bone, heart, kidneys, and skin. Cryptococcal infection has exhibited dormancy and reactivation; therefore, it is most likely that transplant recipients had latent infection before the transplant. Saha et al. have reported that in 52% of the population, there was exposure to *Cryptococcus* before the transplant [8]. Adult and pediatric cryptococcosis in KT recipients exhibit distinct clinical characteristics (Table 1).

**Figure 1:**
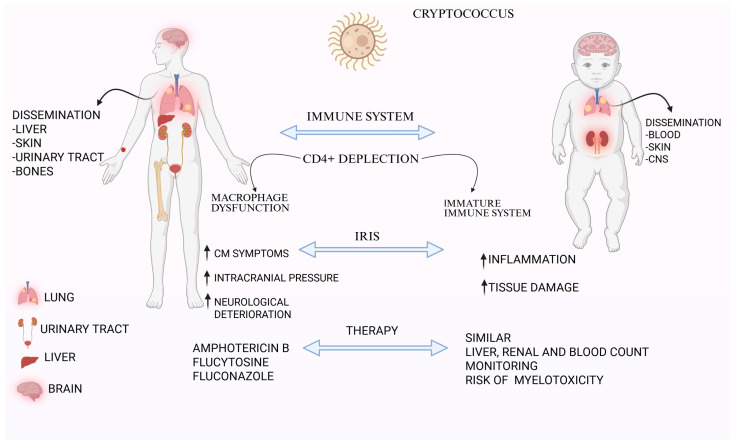
Differences in symptoms and therapeutic management of Cryptococcosis in adults and children. Legend: This figure compares the pathogenesis, clinical manifestations, and treatment considerations of cryptococcosis in adult and pediatric patients. In adults, Cryptococcus often disseminates to the liver, skin, urinary tract, and bones, primarily due to CD4+ T cell depletion and macrophage dysfunction. In children, the immature immune system contributes to dissemination to the blood, skin, and central nervous system (CNS). The immune reconstitution inflammatory syndrome (IRIS) can worsen cryptococcal meningitis (CM) symptoms, increase intracranial pressure, and lead to neurological deterioration in adults, while in children it typically results in heightened inflammation and tissue damage. Therapeutic approaches are similar in both groups, including amphotericin B, flucytosine, and fluconazole. Pediatric management, however, requires careful monitoring for liver, renal, and hematologic toxicity, with particular attention to the risk of myelotoxicity. Created with BioRender.com.

In adult KT recipients, cryptococcosis occurs from months to years post-transplant and most commonly presents as cryptococcal meningitis (CM), a severe CNS infection (Figure 1). Patients commonly present with fever, headache, altered mental status, photophobia, and vomiting [9]. Due to prolonged immunosuppression, symptoms may be insidious, leading to delayed diagnosis and increased mortality risk. CM accounts for 25–72% of cryptococcal infections in this population and is associated with a high mortality rate [10]. Pulmonary cryptococcosis, another common manifestation in adults, often presents asymptomatically or with mild respiratory symptoms such as cough, dyspnea, and chest pain. In some cases, radiological findings reveal solitary or multiple pulmonary nodules, which may be mistaken for malignancy, making early diagnosis challenging and leading to unnecessary interventions [11,12]. In disseminated cryptococcosis, the infection can spread to the skin, bones, liver, and urinary tract, further complicating the disease course [13]. Skin lesions, which may appear as papules, nodules, or ulcers, can be misdiagnosed [14,15] (Figure 1).

Pediatric KT recipients with cryptococcosis tend to exhibit a more variable and less specific clinical presentation compared to adults. The infection in children is rarer and tends to affect the respiratory system more prominently; symptoms may include persistent cough, tachypnoea, fever, and wheezing, which may mimic other respiratory infections, such as tuberculosis and or Pneumocystis jirovecii pneumonia [16]. Chest radiographs and CT scans typically show pulmonary nodules, interstitial infiltrates, or diffuse lung involvement [17]. A noteworthy case described in the literature involves an 18-year-old kidney transplant recipient who underwent transplantation at the age of 12 and subsequently developed cryptococcosis following therapy for acute rejection. The infection was diagnosed based on positive blood cultures and chest tomography findings, which revealed pulmonary nodules [18]. The patient’s history of exposure to pigeon droppings was a potential risk factor [19].

Disseminated cryptococcosis is more common in severely immunosuppressed children, including those on intensive immunosuppressive therapy for acute rejection. The infection can extend to skin, blood, and the CNS. CM in children can present with irritability, lethargy, poor feeding, vomiting, and seizures along with classic adult symptoms of headache and photophobia; children may also present with bulging fontanelles and abnormal reflexes, indicating raised intracranial pressure [20].

Cutaneous involvement in pediatric patients is often more subtle than in adults. Lesions may resemble molluscum contagiosum or wart-like papules, which can lead to misdiagnosis and treatment delays. Due to their developing immune system, children may have a higher risk of rapid fungal dissemination, necessitating aggressive antifungal treatment.

Renal involvement in cryptococcosis is rare but it has been documented in both adults and pediatric patients [21]. Cases of crescentic glomerulonephritis (CGN) associated with cryptococcal infection have been reported in children, suggesting a potential immunological interplay between fungal infection and renal injury [17,22]. In adult patients, histopathological findings range from cryptococcal pyelonephritis to renal cortical involvement with yeast aggregation in the tubules and interstitium [23,24,25].

## 3. Immune Response and Pathogenesis

Both adult and pediatric KT recipients exhibit impaired immune responses to *Cryptococcus neoformans* [26]. CD4+ T cell depletion is a major risk factor, and immunoglobulins and complement proteins contribute to host defense [27]. The encapsulated structure of *Cryptococcus neoformans* serves as a potent immune evasion mechanism, primarily activating the alternative complement pathway, which in turn facilitates opsonization and enhances antigen-specific antibody production [28]. This response is critical for pathogen clearance, yet in immunocompromised individuals, including KT recipients, these mechanisms are often insufficient, leading to fungal dissemination and severe disease manifestations. Effective immunity against *Cryptococcus* relies on recognition and interaction between the fungus and the cells of immune system. It is known that immune response based on Th1-type is associated with protection, whereas Th2-type responses are more associated with worsening of the disease. The internalization of the yeast into phagosome leads to inflammation and production of reactive oxygen and nitrogen species (ROS and RNS). *Cryptococcus neoformans* possesses virulence factors, such as urease and laccase, which manipulate the immune response toward a nonprotective Th2-type [29].

In both adult and pediatric KT recipients, cryptococcosis is primarily associated with impaired cellular immunity, particularly CD4+ T cell depletion. CD4+ T cells play a critical role in coordinating the immune response against *Cryptococcus* by activating macrophages and recruiting other immune cells to control fungal proliferation [30,31]. In adults, prolonged immunosuppressive therapy, including corticosteroids, calcineurin inhibitors (e.g., tacrolimus, cyclosporine), and antiproliferative agents (e.g., mycophenolate mofetil), significantly reduces CD4+ T cell counts and function. This immune dysfunction allows *Cryptococcus* to evade host defenses, leading to widespread dissemination, particularly to the CNS. In pediatric patients, the immune response is further compromised due to the immaturity of the adaptive immune system. Children have lower baseline levels of memory T cells, which limits their ability to mount a rapid and effective secondary immune response upon fungal exposure [32,33]. Additionally, pediatric patients have lower levels of circulating immunoglobulins and a reduced capacity for antigen-specific antibody production, making them more susceptible to disseminated disease.

This could contribute to increased vulnerability in younger patients, particularly those with additional risk factors such as congenital immunodeficiencies or prolonged immunosuppressive therapy.

Macrophage dysfunction is another critical factor in cryptococcosis pathogenesis, affecting both adult and pediatric patients. Macrophages serve as the first line of defense by engulfing *Cryptococcus* through phagocytosis. However, *Cryptococcus neoformans* possesses a polysaccharide capsule that inhibits macrophage activation, reducing the ability of these immune cells to clear the infection. In adult transplant recipients, prolonged exposure to immunosuppressive drugs further suppresses macrophage function, limiting their ability to secrete proinflammatory cytokines such as tumor necrosis factor-alpha (TNF-α), interleukin-6 (IL-6), and interferon-gamma (IFN-γ), which are crucial for antifungal immunity. In pediatric patients, macrophage function is inherently weaker due to incomplete immune system development, leading to inadequate fungal clearance and increased risk of systemic dissemination [34,35].

The complement system plays another vital role in fungal clearance by enhancing phagocytosis and initiating inflammation. In both adults and children, *Cryptococcus* evades the immune response by interfering with complement activation. The alternative complement pathway, which is crucial for innate immune defense, is activated by the fungal capsule. However, excessive complement activation can lead to immune complex deposition, contributing to disease severity in pediatric patients. In contrast, adult transplant recipients often exhibit complement depletion due to chronic immunosuppression, resulting in reduced opsonization and impaired fungal clearance.

Another key difference between adult and pediatric cryptococcosis is the role of immune reconstitution inflammatory syndrome (IRIS). In adult transplant recipients, IRIS occurs when immune function is partially restored, leading to an exaggerated inflammatory response against *Cryptococcus*. This phenomenon is particularly common after tapering immunosuppressive therapy or initiating antifungal treatment. In pediatric patients, IRIS is less frequently observed in children, which can probably be explained by the slower maturation of the immune system, but when it occurs, it can result in severe complications due to excessive inflammation and tissue damage.

In summary, while both adult and pediatric KT recipients exhibit impaired immune responses to *Cryptococcus*, differences in immune system maturity, complement activation, macrophage function, and the risk of IRIS contribute to variations in disease presentation and progression. These insights underscore the need for age-specific antifungal strategies, careful immunosuppressive management, and close monitoring of immune function to improve patient outcomes.

## 4. Laboratory Diagnosis

Diagnosis of cryptococcosis in both adults and pediatric populations relies on a combination of fungal cultures, CrAg detection (serum CrAg sensitivity and specificity are 99.7% (97.4–100) and 94.1% (88.3–98.1), respectively), and cerebrospinal fluid (CSF) analysis (sensitivity and specificity of CSF CrAg are 98.8% (96.2–99.6) and 99.3% (96.7–99.9)) [36]. According to the ECMM and the ISHAM guidelines, lumbar puncture with CSF opening pressure measurement is essential for all patients with suspected CM, as it provides critical diagnostic and prognostic information to guide management and to assess intracranial pressure [7]. The presence of an elevated CSF opening pressure, commonly observed in CM, is associated with poor outcomes and requires prompt therapeutic intervention, such as serial lumbar punctures or, in severe cases, ventriculoperitoneal shunting [37]. While blood and CSF CrAg testing are highly sensitive and specific for cryptococcal disease, additional diagnostic modalities can be particularly useful in pediatric cases. Blood cultures should also be collected in all cases to search for Cryptococcus. In case of pulmonary involvement, visualization of encapsulated yeast forms with narrow budding in the sputum, bronchoalveolar lavage (BAL) or lung tissue biopsy specimens are suggestive of cryptococcal infection. Skin biopsy and PCR for *Cryptococcus* is recommended when there are skin lesions and the suspicion for cryptococcal infection. Urine microscopy and culture have demonstrated utility in detecting *Cryptococcus* in high-risk children, especially those with advanced immunosuppression [38]. The presence of *Cryptococcus* within urinary casts has been reported in a 13-year-old girl with AIDS, emphasizing the potential role of urine analysis in high-risk children and those with suspected disseminated cryptococcosis [21]. This finding highlights the diagnostic value of urine analysis in disseminated disease, particularly in high-risk pediatric patients. Given that pediatric cryptococcosis may present with atypical manifestations, broadening diagnostic considerations is crucial to avoid delays in treatment initiation.

## 5. Imaging and Physical Manifestations

In the era of multidrug-resistant infections, imaging plays a central role in the diagnosis and treatment of cryptococcal infections in KT patients.

Some manifestations of cryptococcal infection in transplant recipients undergoing immunosuppressive treatment may share radiologic features with immunocompetent patients; diagnosis in pediatric patients is sometimes more challenging due to the different clinical signs, so imaging is crucial.

There are no pathognomonic signs in localized and diffuse manifestations of cryptococcal infection and there is often overlap with other pathologies. In such scenarios, knowledge of typical and atypical features of cryptococcal infection allows clinicians to raise suspicion of infectious events and prevent complications and organ damage in a timely manner, a clinical goal, especially in pediatric immunocompromised patients.

As a general rule, localized forms of cryptococcal infection are commonly encountered in immunocompetent patients, while diffuse forms are usually registered in patients with causes of acquired immunodeficiency such as AIDS, as the most common, immunosuppressive drugs, organ transplantation, and lymphoma [39].

### 5.1. Pulmonary Localization

Localization in the lungs is the most frequent form of cryptococcal infection, and the respiratory route is the doorway for systemic infection in immunocompromised patients in the majority of cases.

Chest X-ray examination is sufficient to detect extensive pulmonary involvement by cryptococcosis and is preferable in children with non-complicated disease, but CT represents the gold standard for more complex cases for the finest imaging assessment [39].

MRI is not diffusely employed for lung scans; however, it is employed in selected centers, allowing sufficient evaluation with a radiation dose spare [40].

The main radiographic features of pulmonary cryptococcosis in adults are reported in Table 2.

In a recent systematic review and meta-analysis, Xiong et al. reported the most common CT features identified in the adult population [39]. The most common radiologic signs of infection in immunocompetent patients are bilateral pulmonary solid nodules and masses, that in most of cases are scattered peripherally and do not have predilected lobes. Pulmonary nodules are the result of effective response by the immune system in localizing a fungal infection at its respiratory center; they range from 6 to 10 mm, but sometimes masses with or without lymphadenopathy are detected, requiring differential diagnosis from cancer [41,42]. As a general rule, pulmonary lesions in adults are frequently bilateral both in immunocompetent and immunosuppressed population, however the latter show increased localization to upper lobes. More aggressive forms of infection with *atypical* manifestation are prevalent in the case of immunodeficiency.

Ground-glass opacities, cavitation, and enlarged mediastinal lymphadenopathy are the most documented imaging findings in immunocompromised patients. Explanation of this may be found in a reduced efficiency by the immune system to confine cryptococcal infection. This unrestrained process determines damage to lung tissue resulting in hemorrhagic exudation, visible as a ground-glass opacity or further extending to pulmonary destruction and necrosis with visible cavitations. Mediastinal lymph node enlargement instead follows a lymphatic spread of microorganisms with consequent mediastinal lymphadenitis. Pleural effusion showed low presence (5% of patients). These radiological signs overlap with other pulmonary infectious diseases and malignancies, complicating the diagnosis and delaying treatment. Extensive pulmonary involvement with fungal cavitated nodules does not differ from *Mycobacterium tuberculosis* cases, meaning that radiologic appearance is often misdiagnosed, with a documented percentage of cases of cryptococcal infection mistakenly described as tubercular pneumonia [43].

For these reasons, radiologic evaluation should always be supplemented by laboratory testing to ensure prompt diagnosis and treatment.

Chest radiographic findings between adult and children show a certain overlap; radiographic differences in chest examination are driven largely by host immune response and underlying conditions rather than age per se. It must be noted that while imaging findings in adult population are largely discussed in the literature, fewer literature reports with a restricted number of subjects are available.

In pediatric patients, cryptococcosis is rarer, but often tends to present with a pattern consistent with radiological findings in adults. Immunocompetent patients showed pulmonary localized nodular lesions but cases of more diffuse pulmonary involvement such as masses and consolidation, with variable extension up to the entire lung involvement [16,44].

In a study in the Chinese population on a cohort of 47 pediatric patients, hilar or mediastinal lymphadenopathy were the most common findings (47%) followed by lung nodules (incidence 44.7%) [45].

Studies on the pediatric population confirm the correlation of atypical features, such as cavitation and masses located peripherally, with diffuse cryptococcal infection in immunocompromised patients [46,47]. This general rule, however, finds exception even in the pediatric population; in fact, atypical features like pulmonary cavitation may occur in immunocompetent subjects, probably due to an immature immune response [44,48].

### 5.2. Central Nervous System Localization

The spread of infection over the pulmonary capillary filter brings diffuse disease with hematogenous dissemination. The most common and feared complication of diffuse cryptococcosis is the localization to the CNS. CNS involvement is most commonly reported among adults [49,50,51,52].

Meningoencephalitis is the result of a meningeal colonization through blood–brain barrier diffusion. From meningeal space, infection can spread to the cortex or, more frequently, through perivascular space to basal ganglia.

This event, sometimes fatal, is characteristic of immunocompromised patients, especially those with human immunodeficiency virus (HIV) infection; however, reports in the literature have described meningeal cryptococcosis even in patients with normal immune function [45].

In a population study on adults, Shih et al. reported that the most severe neurological complications occur in immunocompetent patients due to a strong immune response. Comparison among patients with and without T cell response suppression showed how immunocompetent patients presented higher prevalence of seizures and cranial nerve and meningeal signs, with CT findings of meningeal involvement, hydrocephalus, and parenchymal lesions [53].

Rojo-Martin et al. reported two cases of people who presented with neurological symptoms (cephalea, diplopia, speech problems, vomiting, peripheral vertigo). Both had KT less than 20 months prior to admission to hospital and were on immunosuppressive therapy with mycophenolate, tacrolimus, and prednisone. In one case, the cerebral CT scan was normal, while the other presented with slight right-sided occipital hypodensity [49].

Neurologic clinical signs are variable according to the specific localization to the CNS, with symptoms ranging from focal cranial nerve deficiency to headache, fever, and loss of consciousness. Not all CNS localizations present with neurologic signs; in a pediatric population study on 56 patients with CNS invasion, Gao et al. reported that only 58.3% presented neurological symptoms [45].

In the case of CNS infection, CT may be considered for a first line approach, but sensitivity is low, with almost 50% negative scans in the absence of focal intraparenchymal complication [54]. MRI represents, therefore, the method of choice for brain imaging in infectious CNS involvement, with high sensitivity for slight inflammatory changes. The most described manifestations consist in hydrocephalus, ventricular dilatation, focal lesions to brain parenchyma, meningeal localization, and even cerebral vein sinuses thrombosis [45].

Lepto-meningitis or meningoencephalitis is visible at T2 and FLAIR imaging as cortical hyperintensity due to inflammatory edema, while thickening of the meningeal layer is demonstrated with linear contrast enhancement of the meningeal space. Contrast enhancement is, however, related to immune response rather than to fungal invasion [54].

Localization to the basal ganglia, midbrain, thalamus, and hypothalamus may be detected by MRI as a parenchymal localization with a characteristic “hazy brain base” appearance due to dilatation of perivascular Vircow–Robin spaces (VRS) progressively evolving to the formation of pseudocystic lesions in the basal ganglia with the typical T2 and FLAIR “bubble-like” hyperintensity [55]. Other less common areas of localization are the cerebellum and thalamus.

Further spread through VRS leads to intraparenchymal infection, with the formation of inflammatory granulomas leading to microinfarctions in the vascularization territory of perforating arteries, also called *cryptococcomas*. These areas may be visible as hyperintensity on DWI and hypointensity on ADC maps, while contrast-enhanced MRI can show a variable enhancement pattern, with homogeneous enhancement of cryptococcomas or peripheral rim enhancement of necrotic granulomas; due to the inflammatory nature of such lesions, they are more frequent in immunocompetent patients or after antifungal therapy [55].

Ventricular involvement due to intracranial fluid pressure hypertension carries severe neurologic manifestation due to CFS outflow obstruction with hydrocephalus development [56]. The etiology of intracranial hypertension is discussed and both an arachnoid villi drainage impairment due to fungal polysaccharides and macroscopic obstruction of CSF circulation to arachnoid villi have been hypothesized [57]. Ventricular localization has been reported both in pediatric and adult series and also in immunocompetent patients; however, CSF pressure increase may not be accompanied by ventricular dilatation [58].

Probably due to clinical unawareness, some cases of CM may be misdiagnosed as tubercular meningitis; patterns of tubercular meningitis, however, commonly display a higher involvement of basal meningeal space with meningeal enhancement and hydrocephalus [59].

### 5.3. Renal Localization

Renal cryptococcosis (RC) is less common than pulmonary involvement, and aspecific symptoms make diagnosis challenging [60]. Histologically, it is an interstitial infiltration by yeast cells with a variable host immune response. The syndrome has been associated with segmental glomerulosclerosis-like changes with segmental glomerular and interstitial lymphoplasmacytic and granulomatous infiltration, as shown by Ramdial et al. in the case of two pediatric patients with HIV infection [10]. Radiologic findings of renal cryptococcosis are often nonspecific, requiring histopathologic confirmation. As in pulmonary infection, visceral localizations of cryptococcal infection share imaging features in common with other opportunistic infections, such as tuberculosis, fungal pyelonephritis, or HIV-associated nephropathy, making correlation with clinical and laboratory data necessary.

Extensive renal parenchyma involvement may evolve to macroscopic impairment, expressing as pyelonephritic forms, and beyond that to renal papillary necrosis due to destructive necrotizing inflammation [10].

Apart from renal enlargement, ultrasound is rarely effective in detecting parenchymal signs of inflammation, with a known limitation for inflammatory changes; inconstantly it is possible to visualize hypoechoic ill-defined areas. On the other hand, necrotizing evolution with abscess formation and papillary necrosis may be clearly identified using ultrasound imaging [61].

On the contrary, both contrast-enhanced CT and MRI are accurate for the detection of perfusion parenchymal changes due to edema hypoperfusion. Granulomatous inflammation may appear as patchy low-attenuation cortical pyelonephritis-like areas; micro-abscessual evolution is visible as non-enhancing foci with variable extent on post-contrast imaging.

### 5.4. Lymphatic Localization

Hylar and mediastinal lymphonodal involvement is a common finding in association with pulmonary involvement, as previously discussed.

Other thoracic localization involves axillary nodes with palpable lymphadenopathy.

Abdominal spread of infection can cause lymphadenopathy, localized in retroperitoneal space and mesenteric and celiac regions [45].

Splenic invasion is also documented, with splenomegaly and parenchymal hypodense lesions [45].

### 5.5. Hepatobiliary Localization

Among the rare localizations of *Cryptococcus* infection, hepatobiliary localization is characterized by relevant clinical manifestations and radiological findings, showing hepatomegaly with localized parenchymal nodules [45].

Extrahepatic biliary duct infiltration has also been reported [62]. No substantial differences have been found between adult and pediatric patients; in fact, specific symptoms such as jaundice and urinary and bowel dysfunction, associated with systemic signs and positive laboratory values, suggest hepatic impairment with biliary obstruction [63]. In such cases, the imaging modalities of choice are ultrasound and MRI in conjunction with MR-cholangiography. Both may show hepatomegaly, low density lesions to liver parenchyma, and, in the case of biliary involvement, signs of intrahepatic bile duct dilatation, diffuse thickening of the walls of the common bile duct, and narrowing of the lumen. Other MR signs include periductal soft tissue thickening, with decreased intensity on T2-weighted imaging due to inflammatory infiltration [64].

### 5.6. Cutaneous Localization

Cutaneous cryptococcosis has been frequently reported in renal transplant recipients. Cutaneous cryptococcosis, due to hematogenous spread, is reported in around 20% of patients with disseminated cryptococcosis and it may manifest with almost any type of the cutaneous lesion (papule, nodules, ulcerations…). Given this, it is easy to misdiagnose it, as it can mimic other skin diseases. It is therefore important to consider cryptococcosis, especially in transplant recipients, when presented with cutaneous lesions and signs of disseminated cryptococcosis, although cutaneous cryptococcosis appears to be more frequent in liver transplant recipients. Nowak et al. reported a case of a 65-year-old patient who had received KT and was under immunosuppressive therapy and presented erythematous, solid, regular, and well-defined papules on the skin of the face as well as signs of disseminated cryptococcosis. The patient underwent graft nephrectomy and antifungal therapy with liposomal B amphotericin plus flucytosine. Despite antifungal therapy, the woman died due to cryptococcosis disseminated to other organs, such as the thyroid [65].

Another case of cutaneous cryptococcosis is reported by Song et al., who describe the case of a 43-year-old male who had received a KT and was on immunosuppressive therapy with tacrolimus, mycophenolate, and cyclosporine. The man presented progressively worsening skin lesions, which then evolved in painful, inflamed, diffuse, erythematous, and indurated lesions on both legs and ulcerations. The progression of disease led to systemic symptoms, signaling that the infections had disseminated to other organs as well. PCR performed on skin biopsy revealed the presence of *Cryptococcus neoformans* and the man was successfully treated with amphotericin B lipid complex, then switched to fluconazole [15].

Diagnosis of cutaneous cryptococcosis requires skin biopsy and microscopic evaluation. Mucoid colonies are typical of the fungus and sometimes round, encapsulated yeast-like budding cells can be observed, representing *Cryptococcus neoformans* itself.

## 6. Management

Therapeutic strategies are broadly similar in both populations but require weight-based dose adjustments and meticulous and careful pharmacokinetic monitoring, as well as consideration of drug-related toxicities to minimize toxicity while ensuring efficacy in pediatric patients [66]. The first-line antifungal therapy for CM in immunocompromised patients includes liposomal amphotericin B (AmB) (3–4 mg/kg/day) plus flucytosine (25 mg/kg every 6 h) for at least two weeks, followed by fluconazole (400–800 mg/day for eight weeks), and subsequent maintenance with fluconazole 200 mg/day for 12 months [7]. AmB (5 mg/kg/day) and fluconazole (12 mg/kg/day) are the mainstays of treatment, but potential nephrotoxicity must be considered, particularly in the context of renal transplantation [67]. A reduction in immunosuppressive therapy is critical but must be balanced against the risk of graft rejection [68]. In pediatric patients, it is preferable to use specified dose adjustments to ensure safety and efficacy: AmB (1 mg/kg per day IV) plus flucytosine (100 mg/kg per day orally in 4 divided doses) for 2 weeks (for the non–HIV-infected, non-transplant population, follow the treatment length schedule for adults), followed by fluconazole (10–12 mg/kg per day orally) for 8 weeks. For AmB-intolerant patients, either liposomal AmB (5 mg/kg per day) or AmB lipid complex (5 mg/kg per day) treatment is advised. Maintenance therapy is fluconazole (6 mg/kg per day orally) [2].

A major challenge in the management of cryptococcosis across age groups is C-IRIS, a paradoxical worsening of symptoms driven by an exaggerated inflammatory response upon immune recovery [69]. C-IRIS is particularly concerning in HIV-positive individuals initiating antiretroviral therapy and in SOT recipients undergoing immunosuppressing tapering. The condition manifests as worsening CM symptoms, increased intracranial pressure, and, in severe cases, neurological deterioration.

ECMM/ISHAM guidelines recommend corticosteroid therapy (prednisone 0.5–1 mg/kg/day for 4–6 weeks) for moderate-to-severe C-IRIS cases, particularly in transplant recipients [7]. However, prolonged corticosteroid use in immunocompromised individuals carries risks, including heightened susceptibility to secondary infections and metabolic complications, necessitating careful patient monitoring. In KT recipients, the risk of C-IRIS underscores the need for gradual immunosuppressant tapering rather than abrupt dose reductions, as well as close clinical and radiological surveillance [70]. Close monitoring of antifungal drug interactions with immunosuppressants, such as tacrolimus and cyclosporine, is essential to prevent toxicity or reduced efficacy.

Overall, a multidisciplinary approach involving transplant specialists, infectious disease experts, and critical care physicians is essential in optimizing the diagnosis and management of cryptococcosis in both adult and pediatric KT recipients. With early detection, tailored antifungal therapy, and careful immunosuppressive adjustment, outcomes can be significantly improved in high-risk populations.

## 7. Conclusions

Cryptococcosis in KT patients remains a formidable challenge, requiring early recognition and individualized treatment. While there are substantial overlaps in presentation and management between adult and pediatric patients, key differences exist in clinical manifestations, immune responses, and therapeutic considerations. Global guidelines now emphasize the importance of screening high-risk transplant recipients, optimizing antifungal therapy, and carefully adjusting immunosuppressive regimens to improve patient outcomes. By understanding these nuances, clinicians can improve outcomes in both populations and tailor interventions accordingly.

## Figures and Tables

**Table 1:** Comparison of clinical manifestations and risk factors for cryptococcosis in adult and pediatric kidney transplant recipients.

Feature	Adult KT Recipients	Pediatric KT Recipients
Primary Clinical Presentation	Meningitis, fever, headache, altered mental status	Pulmonary involvement, disseminated disease
Prevalence of Pulmonary Involvement	Often asymptomatic or mild	More common, nodules and interstitial infiltrates on imaging
Renal Involvement	Rare, includes pyelonephritis and cortical involvement	Rare, includes crescentic glomerulonephritis
Risk Factors	Chronic immunosuppression, history of rejection therapy	Severe immunosuppression, environmental exposure

**Table 2:** Radiographic features of pulmonary cryptococcosis.

Main Features	Atypical Features
Multiple nodules	Cavitation
Segmental/lobar consolidation	Pleural effusion
Reticular interstitial thickening	Lymphoadenopathy
	Miliary disease
	Ground glass/Halo Sign
